# Epidermal stem cell-derived exosomes improve wound healing by promoting the proliferation and migration of human skin fibroblasts

**DOI:** 10.1093/burnst/tkae047

**Published:** 2024-12-16

**Authors:** Deni Kang, Xiaoxiang Wang, Wentao Chen, Lujia Mao, Weiqiang Zhang, Yan Shi, Julin Xie, Ronghua Yang

**Affiliations:** Department of Burn and Plastic Surgery, Guangzhou First People's Hospital, Guangzhou Medical University, 1 Panfu Road, Yuexiu District, Guangzhou City, Guangdong Province, 510180, China; Department of Burns, The First Affiliated Hospital of Sun Yat-Sen University, 58 Zhongshan Second Road, Yuexiu District, Guangzhou City, Guangdong Province, 510062, China; Department of Medical cosmetology, Foshan Second People's Hospital, 78 Weiguo Road, Chancheng District, Foshan City, Guangdong Province, 528000, China; Department of Burn and Plastic Surgery, Guangzhou First People's Hospital, South China University of Technology, 1 Panfu Road, Yuexiu District, Guangzhou City, Guangdong Province, 510180, China; The First Clinical Medical College, Guangdong Medical University, 2 Wenming East Road, Xiashan District, Zhanjiang City, Guangdong Province, 524002, China; Department of Plastic, Medical Center of Burn Plastic and Wound Repair, The First Affiliated Hospital, Jiangxi Medical College, Nanchang University, No. 17 Yongwai Zheng Street, Donghu District, Nanchang City, Jiangxi Province, 330006, China; Department of Burns, The First Affiliated Hospital of Sun Yat-Sen University, 58 Zhongshan Second Road, Yuexiu District, Guangzhou City, Guangdong Province, 510062, China; Department of Burn and Plastic Surgery, Guangzhou First People's Hospital, Guangzhou Medical University, 1 Panfu Road, Yuexiu District, Guangzhou City, Guangdong Province, 510180, China

**Keywords:** Epidermal stem cells, Exosomes, Wound healing, Human skin fibroblasts, Skin regeneration, Migration, Proliferation

## Abstract

**Background:**

Epidermal stem cells (ESCs) are primarily located in the basal layer of the epidermis and play a crucial role in wound healing. ESCs-derived exosomes (ESCs-Exo) are emerging as promising candidates for skin regeneration and wound healing. However, the underlying mechanisms remain unclear. This study aims to investigate the role and mechanisms of ESCs-Exo in promoting the proliferation, migration, and collagen synthesis of human skin fibroblasts (HSFBs).

**Methods:**

This study generated, isolated, and characterized ESC-Exos. The effects of ESCs-Exo on the proliferation of human skin fibroblasts (HSFBs) were detected via Cell Counting Kit-8 (CCK8), 5-Ethynyl-2'-deoxyuridine (EdU), and Proliferating Cell Nuclear Antigen (PCNA) and Marker of Proliferation Ki-67 (MKI67) gene expression methods. The effect of ESCs-Exo on the migration of HSFBs was detected via a transwell assay and a scratch test. The concentrations of collagen secreted by the HSFBs and the mRNAs of the two kinds of collagen expressed by the HSFBs were analyzed. We also analyzed the phosphorylation of Protein Kinase N1 (PKN1) and the expression of cyclins via western blotting. Finally, the effect of ESCs-Exo on wound healing was verified by animal experiments, and the key genes and signaling pathways of ESCs-Exo were excavated by transcriptomic analysis.

**Results:**

Western blotting revealed that the exosomes of ESCs highly expressed established markers such as Alix, CD63, and CD9. ESC-Exos significantly promoted HSFB proliferation and migration in a dose-dependent manner, as well as HSFB collagen synthesis, and effectively increased the ratio of collagen III/I. In addition, bioinformatics analysis showed that the expression of key gene C-X-C motif chemokine ligand 9 was lower in the ESCs-Exo group, which may promote wound healing by regulating PKN1-cyclin and tumor necrosis factor signaling pathways. Animal experiments demonstrated that ESCs-Exo could reduce inflammation and accelerate wound healing.

**Conclusions:**

In this study, we found that ESCs-Exo may improve wound healing by promoting the proliferation and migration of HSFBs.

Highlights

- The mechanism by which ESCs-Exo accelerate wound healing may involve promoting the proliferation and migration of HSFBs.
- ESCs-Exo may regulate inflammatory and immune responses by modulating PKN1-cyclin to promote HSFBs proliferation and modulate tumor necrosis factor signaling pathways.
- We found that treating HSFBs with ESCs-Exo effectively increased the ratio of collagen III/I, promoting the formation of scar-free wounds.

## Background

The skin is the largest organ of the human body and is responsible for the protection of the body, temperature regulation, and other functions [1]. It is the most vulnerable organ, and external factors such as burns and trauma can cause skin damage. Once the skin is damaged, a series of intracellular and intercellular mechanisms are triggered to repair tissue damage. The healing of skin wounds occurs through the inflammatory response period, hyperplasia period, remodeling period, and other processes, which are complex processes that require the participation of various cells [2]. Many internal and external factors can affect the process of wound healing, such as the age of the body, nutritional status, accompanying underlying diseases, the area of injury, the depth of injury, and wound infection. At present, there are many patients with car accident injuries, burns, chronic refractory wounds, and war wounds in clinical practice. Therefore, it is very important to study the cellular and molecular mechanisms related to wound healing.

The main functional cells involved in wound healing and scar remodeling are fibroblasts, which proliferate, migrate to the wound, and secrete collagen fibers to seal and protect the wound [3]. Therefore, studying the proliferation and migration of fibroblasts for wound healing is highly important.

Previous studies have shown that epidermal stem cell (ESC) transplantation can promote wound healing [4,5]. However, the specific mechanisms involved remain underexplored. Moreover, low transplantation efficiency, tumor formation, inadequate cell number availability, immune rejection, and ethical conflicts have complicated efforts to evaluate ESC treatment in clinical trials [6,7]. A recent study revealed that the therapeutic effects of stem cell treatment may be achieved mainly through exosome-mediated mechanisms [8], and exosome therapy may be a promising and technically advantageous alternative to stem cell treatment [9]. Exosomes are extracellular vesicles 50–150 nm in diameter that are protected by a phospholipid bilayer membrane and carry proteins, lipids, and nucleic acids, including MicroRNAs (miRNAs), which can be transmitted to the wound area without degradation [10]. Unfortunately, unlike mesenchymal stem cell-derived exosomes (MSC-Exos) and adipose stem cell-derived exosomes (ASC-Exos), the mechanism by which ESC-derived exosomes (ESCs-Exo) promote skin regeneration during wound healing has not been investigated.

We wondered whether ESC-Exos could stimulate the proliferation and migration of skin fibroblasts and by what mechanism these exosomes exert this effect. In this study, we efficiently generated, isolated, and characterized ESCs-Exo and explored their optimal concentration and functions via *in vitro* assays of differentiation, migration, and proliferation. Our findings show that ESCs-Exo can promote the proliferation and migration of human skin fibroblasts (HSFBs) and that 40 μg/ml could be the optimal concentration for the beneficial effects of ESCs-Exo. In addition, ESCs-Exo may promote HSFB proliferation through PKN1-cyclin signaling.

## Methods

### ESC isolation and identification

Fresh human skin tissue was collected from blepharoplasty and skin removal patients at the burn and skin surgery clinic of our hospital. The specimens were collected with written consent and signed informed consent before surgery (three men and three women, aged 18–50 years). Briefly, the skin tissues were incubated with 0.25% dispase II (Sigma, St. Louis, USA) overnight at 4°C. The epidermis was minced and digested with 0.05% trypsin at 37°C for 10–15 min. The keratinocyte cell suspension was filtered, washed with phosphate-buffered saline (PBS), and resuspended in keratinocyte growth medium 2 (PromoCell, Heidelberg, Germany) supplemented with 0.125 ng/ml epidermal growth factor, 4 μl/ml bovine pituitary extract, 5 μg/ml insulin, 0.33 μg/ml hydrocortisone, 0.39 μg/ml epinephrine, 10 μg/ml transferrin, 0.06 mM CaCl_2_, and 100 U/ml streptomycin and penicillin and seeded in culture dishes coated with type IV collagen (Sigma). After incubation at 37°C for 10 min, the nonadherent cells were rinsed off with PBS. The adherent ESCs were cultured in keratinocyte growth medium 2 supplemented with 10 μM Y-27632 (STEMCELL Technologies, Aldrich, USA) to promote ESC expansion and inhibit ESC differentiation [11–13]. The levels of CD71 expression and α6 integrin expression were detected via flow cytometry.

### ESC-Exo collection and quantification

Briefly, the cells were grown in 10–15 cm^2^ flasks under normal culture conditions in media supplemented with 10–15% fetal bovine serum (FBS) until they reached a confluency of 70–80%. Thereafter, the cells were cultured for >48 h in medium supplemented with extracellular vesicle-free FBS (A27208, Gibco; FBS was depleted of bovine extracellular vesicles by ultracentrifugation at 110000 g for 180 min). The culture medium was collected and centrifuged at 300 g at 4°C for 10 min and 2000 g at 4°C for 20 min to remove dead cells. The supernatants were further centrifuged at 10 000 g for 30 min at 4°C to eliminate contaminating cellular debris. The extracellular vesicles were pelleted from the final supernatants by ultracentrifugation for 70 min at 4°C and 110 000 g using an SW 32 Ti swinging bucket rotor (Beckman Coulter, Fullerton, CA, USA). The pellets were washed in PBS to eliminate contaminating proteins. Another round of centrifugation was performed at high speed, and the supernatants were discarded. The extracellular vesicle samples were used for transmission electron microscopy and western blot analysis.

### HSFB isolation

The dermal tissue was cut into chyme and centrifuged at 200 g for 5 min. The mixture was discarded. The tissue blocks were planted in a 25 cm^2^ culture bottle with a blowpipe and placed upside down for 2–4 h in an incubator with 5% CO_2_ at 37°C and saturated humidity. Low-sugar medium containing 10% FBS in Dulbecco's Modified Eagle Medium (DMEM) was added for culture. After 3–5 days, the medium was changed for the first time according to the degree of cell adhesion. After 5–7 days of culture, fusiform primary dermal fibroblasts were observed. The medium was changed every 2–3 days, after the plants completely climbed. After the cells reached 80–90% confluence, the culture medium was removed, and the cells were washed with aseptic PBS. The digested cells were then transfused with 0.25% trypsin (containing 0.02% Ethylenediaminetetraacetic Acid (EDTA)) (~2–3 ml in a 25 cm^2^ culture bottle) or frozen at 37°C.

### HSFBs proliferation and migration

HSFBs were treated with 20 or 40 μg/ml exosomes for 24 h and then subjected to EdU and diamidinyl phenyl indole (DAPI) staining for the analysis of cell proliferation. The 0 μg/ml exosome treatment was used as a control, and the number of EdU-positive cells was measured; *n* = 6 for each group, and the experiments were repeated three times to confirm the results. Moreover, cell proliferation was measured by measuring the mRNA expression of PCNA and MKI67 in the HSFBs; *n* = 3 for each group, and the experiments were repeated three times to confirm the results.

HSFBs were treated with 20 or 40 μg/ml exosomes for 48 h, and migration was tested via a transwell assay. The 0 μg/ml exosome treatment was used as a control, and the relative migration ratio was normalized to that of the control. The migration of cells at 24 and 48 h was measured via a wound-scratch assay. The migration ratios of the cells at 24 and 48 h were calculated; *n* = 6 for each group, and the experiments were repeated three times to confirm the results.

### Collagen synthesis in HSFBs

First, HSFBs was seeded in 6-well plates, and 2.5 ml of DMEM containing 10% FBS was added to each well. After the HSFBs were cultured for 72 h, they reached 80% confluence. HSFBs were treated with 20 or 40 μg/ml exosomes for 72 h. Afterward, the conditioned medium was collected, and collagen type I and III levels were quantified via enzyme-linked immunosorbent assay (ELISA) kits (Abcam).

### Phosphorylation of PKN1 and expression of cyclins

HSFBs were treated with 20 or 40 μg/ml exosomes for 72 h, and the mRNA expression levels of Cyclin D1 (CCND1), CCNE1, and CCNA2 in HSFBs were analyzed via quantitative real-time polymerase chain reaction (qRT-PCR). The 0 μg/ml exosome treatment was used as a control, and the expression levels were normalized to those of the control. The protein expression levels of p-PKN1, PKN1, cyclin D1, cyclin E1, and cyclin A2 were analyzed via western blotting. Glyceraldehyde-3-Phosphate Dehydrogenase (GAPDH) was used as a loading control. The 0 μg/ml exosome treatment was used as a control, and the expression levels were normalized to those of the control; *n* = 3 for each group, and the experiments were repeated three times to confirm the results.

### qRT-PCR

Total RNA was extracted via TRIzol. The RNA concentration and purity were determined by measuring the optical density at 260 and 280 nm. Real-time PCR amplification was performed in triplicate. Quantitative PCR was conducted via SYBR Green RT-PCR (Takara Biotechnology Co., Ltd, Tokyo, Japan) to generate cDNA fragments. Relative mRNA expression levels were calculated via the 2^-ΔΔCt^ method and normalized to actin expression.

### Western blotting

Total protein was extracted from fibroblasts via radioimmunoprecipitation assay buffer supplemented with a protease inhibitor cocktail (Beyotime, China). The protein concentration was measured via a Bicinchoninic Acid (BCA) protein assay kit (Thermo Fisher Scientific). After the protein concentration was determined, equal amounts of protein (20 μg) were separated via 10% Sodium Dodecyl Sulfate-Polyacrylamide Gel Electrophoresis (SDS-PAGE) and subsequently transferred onto polyvinylidene fluoride membranes. The membranes were blocked with 5% Bovine Serum Albumin (BSA) and incubated overnight at 4°C with primary antibodies. The next day, secondary antibodies were applied at room temperature for 1 h. The polyvinylidene fluoride membranes containing the target proteins were visualized via an AI800 imaging system (GE, USA). Quantitative analysis was carried out via ImageJ software.

### Immunofluorescence assay

For immunocytochemistry, fibroblasts were initially fixed with 4% paraformaldehyde for 30 min and then washed three times with PBS. The cells were permeabilized at room temperature in 0.1% Triton X-100/TBST for 30 min. Subsequently, a 5% BSA blocking solution was applied to block nonspecific antigenic sites, and the cells were incubated at room temperature for 30 min. The membranes were then incubated with primary antibodies overnight at 4°C. Next, the cells were incubated with a horseradish peroxidase-conjugated secondary antibody (goat anti-rabbit, Affinity, 1 : 500) for 1 h. The cells were then stained with DAPI for 10 min (Servicebio, China). Finally, images were captured via a confocal microscope (Zeiss, Oberkochen, Germany).

### Section staining

The tissue sample was sliced and fixed on a slide, dewaxed in warm water and dehydrated with ethanol. The slices are then dyed with Weigert’s iron hematoxylin solution, hematoxylin solution, and phosphotungstic acid solution to wash away excess dye. Finally, the mixture was dehydrated, made transparent, and sealed with neutral gum. After drying, the results were observed under a microscope and recorded.

### ELISA

The samples were either stored at −80°C or immediately analyzed via human ELISA kits for interleukin (IL)-1β, tumor necrosis factor (TNF)-α, and IL-10. All procedures were conducted according to the manufacturer’s instructions, and the absorbance was measured at 450 nm via a microplate reader (Bio-Rad, USA). Each measurement represents the average of at least three independent experiments.

### Animal studies

Healthy male Standard Deviation (SD) rats (6–8 weeks of age, 200–250 g) were purchased from Guangzhou Ruige Biotechnology Co., Ltd (Guangzhou, China) for this experiment. All experiments involving rats were approved by the Animal Protection and Utilization Committee of Guangzhou First People’s Hospital. The mice were randomly divided into two treatment groups: (1) the control group (PBS) and (2) the Exo group (40 μg/ml). There were six animals per experimental group. The mice were observed for 1 week before the experimental procedure began. After being anesthetized with 0.3% phenobarbital sodium (0.1 ml/10 g) by intraperitoneal injection, two 10 mm circular skin wounds were formed on the backs of the mice. Four points above and below the wound, with an average injection of 10μl at each point and 10μl at the center of the wound for 3 consecutive days. At the end of the experiment, the wound of the mouse was covered with dressing and the mouse was euthanized with cervical dislocation.

### Statistical analysis

The data were analyzed via PRISM 8.0 software (GraphPad, San Diego, CA, USA). The values are expressed as the means ± standard deviation (SD). Differences among experimental groups were analyzed by Student’s t test (for single comparisons), one-way Analysis of Variance (ANOVA), and two-way ANOVA (or a mixed model). All the experiments were performed at least three times. All the statistical analyses were performed with Minitab software (Minitab, State College, PA, USA) and are presented as ^*^P < 0.05, ^*^^*^P < 0.01, ^*^^*^^*^P < 0.001, or ^*^^*^^*^^*^P < 0.001.

**Figure 1:**
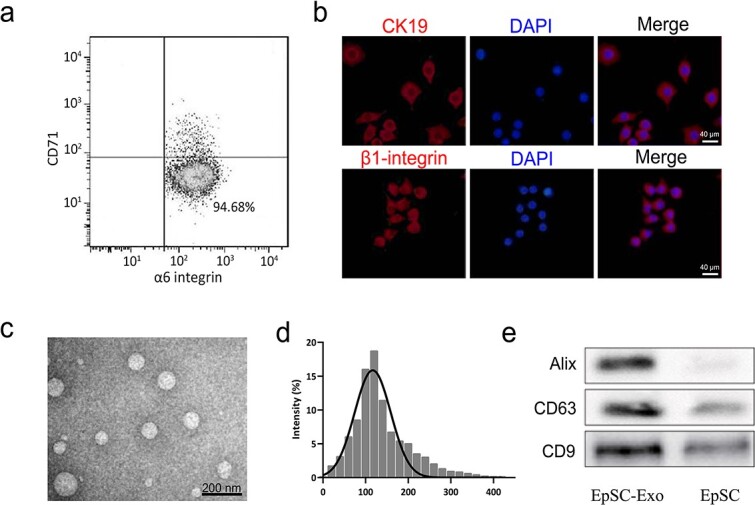
Identification of human ESCs and their derived exosomes. (**a,b**) Biomarkers of ESCs were detected via flow cytometry and immunofluorescence staining, respectively. Scale bar: 40 μm. (**c**) Identification of exosomes via transmission electron microscopy. (**d**) Size (in nm) of the particles in the isolated exosome mixture obtained through nanoparticle tracking analysis. (**e**) Detection of Alix, CD63, and CD9 expression by western blot analysis. *ESCs* pidermal stem cells

**Figure 2:**
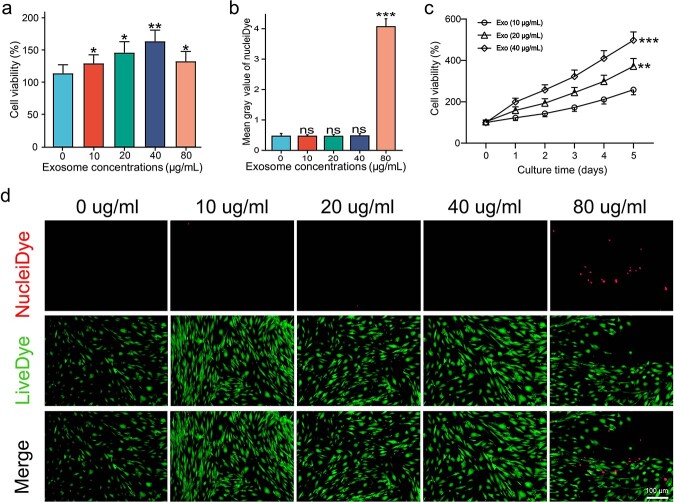
Optimum ESCs-Exo concentration in HSFBs culture. (**a**) HSFBs were treated with different concentrations of exosomes for 24 h and cell viability was determined via a CCK8 assay. Scale bar: 50 μm. (**b,d**) HSFBs were treated with different concentrations of exosomes for 24 h, and cell viability was determined via a live/dead assay. Green, live cells; red, dead cells. Scale bar: 100μm. (**c**) HSFBs were treated with 20 or 40 μg/ml exosomes for different durations and cell viability was determined via a CCK8 assay. The 0 μg/ml exosome treatment was used as a control. The data are presented as the mean ± SD. ^*^p < 0.05, ^*^^*^p< 0.01, ^*^^*^^*^p < 0.001 compared with the control; ns, not significant. *ESCs* pidermal stem cells，*HSFBs* human skin fibroblasts

## Results

### Characterization of ESCs and their exosomes

Primary ESCs were isolated from the skin of patients who underwent double-upper-eyelid operation at our hospital. Flow cytometry analysis revealed that ESCs expressed low levels of CD71 and high levels of α6 integrin (Figure 1a). Immunofluorescence staining revealed that these cells also expressed high levels of β1 integrin and CK19, two other biomarkers of ESCs (Figure 1b). The vesicle morphology of the exosomes was confirmed via transmission electron microscopy (Figure 1c), and their size and concentration were quantified via nanoparticle tracking analysis (Figure 1d). ESC-Exos diameters ranged from 20–400 nm, with peaks at 120 nm, which was consistent with previously reported exosome size distributions [14]. Western blotting revealed that the exosomes of ESCs highly expressed established markers such as Alix, CD63, and CD9 (Figure 1e). These results demonstrated that the isolated ESCs-Exo met the general standards of the definition of exosomes.

### Optimum ESCs-Exo concentration in HSFB culture

To investigate the optimum concentration of ESCs-Exo in HSFB culture, different concentrations of ESCs-Exo were added to the culture medium. As shown in Figure 2a, after a 24-h incubation period, ESCs-Exo significantly increased the proliferation of HSFBs in a dose-dependent manner, reaching a plateau at 40 μg/ml. Figure 2b and c show little cytotoxicity when the concentration of ESCs-Exo was <80 μg/ml. Figure 2c shows that ESCs-Exo significantly increased the viability of HSFBs in a concentration-dependent manner. These findings indicate that 40 μg/ml could be the optimal concentration for the beneficial effects of ESCs-Exo. However, to further support this claim and make the subsequent experiments more convincing, we selected both 20and 40 μg/ml for all further experiments.

### ESC-Exos promote the proliferation of HSFBs

**Figure 3:**
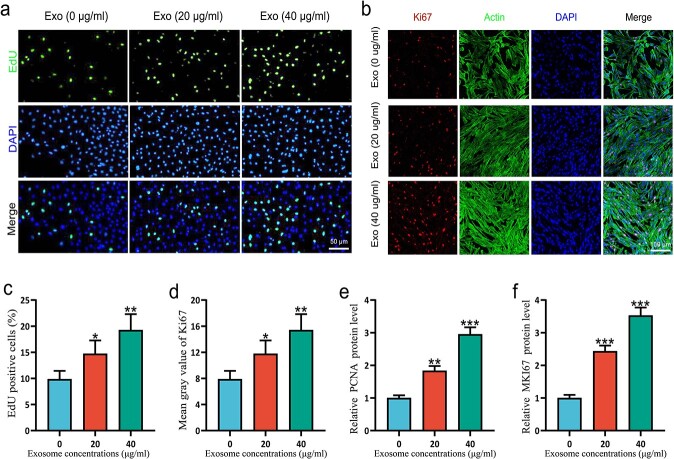
ESCs-Exo promoted the proliferation of HSFBs. (**a,c**) HSFBs were treated with 20 or 40 μg/ml exosomes for 24 h and then subjected to EdU and DAPI staining for the analysis of cell proliferation. Scale bar: 50μm. (**b,d**) Ki-67, actin, and DAPI staining for the analysis of cell proliferation. Scale bar: 100μm. (**e,f**) Cell proliferation was measured by measuring the mRNA expression of PCNA and MKI67, respectively, in HSFBs. The data are presented as the mean ± SD. ^*^p < 0.05, ^*^^*^p < 0.01, ^*^^*^^*^p < 0.001 compared with the control. *ESCs* pidermal stem cells,*HSFBs* human skin fibroblasts

To investigate the effect of ESCs-Exo treatment on proliferation, different concentrations of ESCs-Exo were added to the culture medium, whereas 0 μg/ml exosome treatment was used as a control. As shown in Figure 3a and c, after a 24-h incubation period, ESC-Exos significantly increased the proliferation of HSFBs. These findings were corroborated by staining for the proliferative markers EdU [15] (Figure 3b) and Ki-67 [16] (Figure 3d). Moreover, cell proliferation was measured by measuring the mRNA expression of PCNA [17] (Figure 3e) and MKI67 [18,19] (Figure 3f) from the HSFBs, and the same results were obtained. The experimental results show that ESCs-Exo promote the proliferation of HSFBs.

### ESC-Exos promote the migration of HSFBs

**Figure 4:**
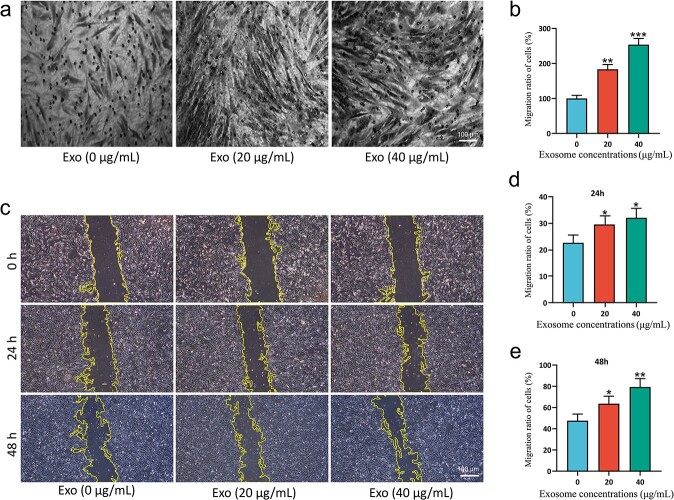
ESCs-Exo promoted the migration of HSFBs. (**a,b**) HSFBs were treated with 20 or 40 μg/ml exosomes for 48 h and migration was tested via a transwell assay. The 0 μg/ml exosome treatment was used as a control and the relative migration ratio was normalized to that of the control. Scale bar: 100μm. (**c**) HSFBs were treated with 20 or 40 μg/ml exosomes. The migration of cells at 24 and 48 h was measured via a wound-scratch assay. Scale bar: 100μm. (**d,e**) Migration ratios of the cells at 24 and 48 h were calculated. The data are presented as the mean ± SD. ^*^p < 0.05, ^*^^*^p < 0.01, ^*^^*^^*^p< 0.001 compared with the control. *ESCs* pidermal stem cells, *HSFBs* human skin fibroblasts

To determine the effect of ESCs-Exo on migration, we first utilized a transwell assay to examine the migration of HSFBs. As shown in Figure 4a and B, ESCs-Exo strongly promoted the migration of HSFBs at a concentration of 40 μg/ml. Moreover, we measured 24- and 48-h distances traveled by mitotically inactivated HSFBs via a standard scratch-wound assay; 0 μg/ml exosome treatment was used as a control. As shown in Figure 4c–e, ESCs-Exo greatly enhanced the movement of HSFBs in a dose-dependent manner.

### ESC-Exos promote collagen synthesis in HSFBs

**Figure 5:**
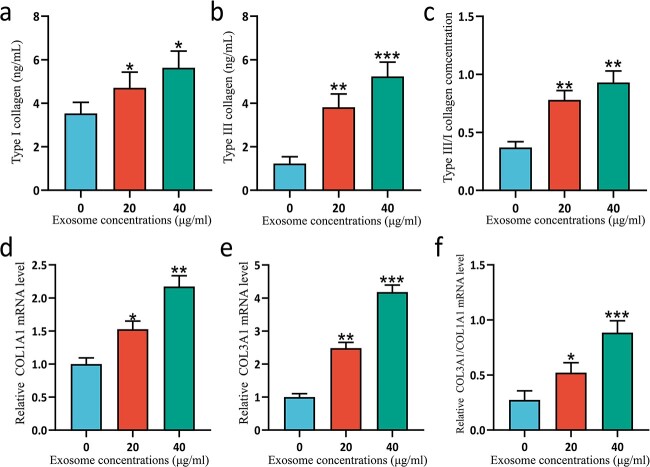
ESCs-Exo promoted collagen synthesis in HSFBs. HSFBs were treated with 20 or 40 μg/ml exosomes for 72 h and the secretion of collagen I (**a**) and collagen III (**b**) was analyzed via ELISA. The ratio of the collagen III/I concentration was calculated (**c**). *n* = 6 for each group and the experiments were repeated three times to confirm the results. Moreover, the mRNA expression levels of COLA1A (**d**) and COL3A1 (**e**) in HSFBs were analyzed via qRT-PCR. The 0 μg/ml exosome treatment was used as a control and the expression levels were normalized to those of the control. The ratio of COL3A1/COLA1A mRNA expression was also compared (**f**). *n* = 3 for each group and the experiments were repeated three times to confirm the results. The data are presented as the mean ± SD. ^*^P < 0.05, ^*^^*^P < 0.01, ^*^^*^^*^P < 0.001 compared with the control. *ESCs* pidermal stem cells, *HSFBs* human skin fibroblasts

To evaluate the antiscarring ability of ESCs-Exo, collagen I and collagen III synthesis in the HSFBs was measured. The collagen III/I ratio in normal adult skin ranges from 3.5 : 1 to 6 : 1, whereas in keloids, the ratio is 1 : 19. Our experiments demonstrated that treating HSFBs with ESCs-Exo effectively increased the ratio of collagen III/I at both the protein (Figure 5a–c) and gene (Figure 5d–f) levels. Exosomes isolated from ESCs have potential antiscarring effects.

### ESCs-Exo promote the phosphorylation of PKN1 and the expression of cyclins

**Figure 6:**
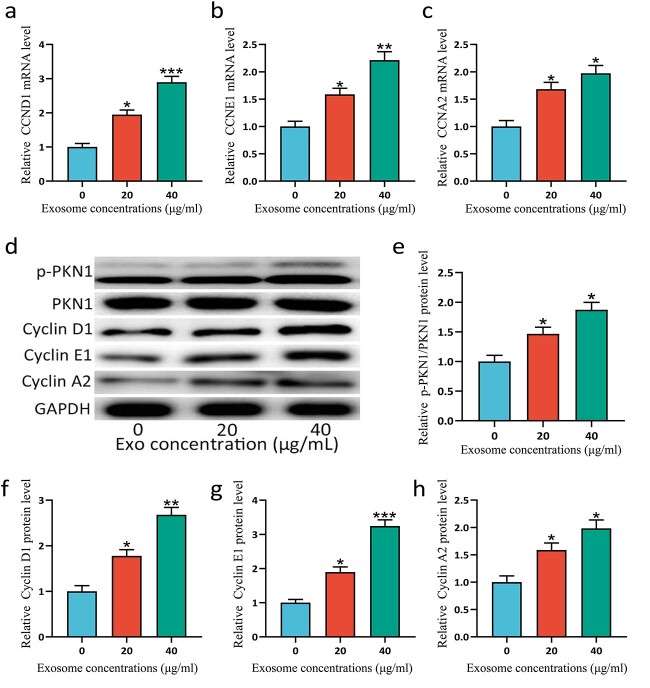
ESCs-Exo promoted the phosphorylation of PKN1 and the expression of cyclins. HSFBs were treated with 20 or 40 μg/ml exosomes for 72 h and the mRNA expression levels of CCND1 (**a**), CCNE1 (**b**), and CCNA2 (**c**) in HSFBs were analyzed via qRT-PCR. The 0 μg/ml exosome treatment was used as a control and the expression levels were normalized to those of the control. (**d**) Protein expression levels of phosphorylated (p)-PKN1, PKN1, cyclin D1, cyclin E1, and cyclin A2 were analyzed via western blotting. GAPDH was used as a loading control. The 0 μg/ml exosome treatment was used as a control and the expression levels were normalized to those of the control (**e-h**). *n* = 3 for each group, and the experiments were repeated three times to confirm the results. The data are presented as the mean ± SD. ^*^p < 0.05, ^*^^*^p < 0.01, ^*^^*^^*^p < 0.001 compared with the control. *ESCs* pidermal stem cells, *HSFBs* human skin fibroblasts

PKN1 has been reported to regulate the proliferation of cells [20–22], while cyclins also play a central role in regulating proliferation [23,24]. PKN1 plays a critical role in vascular wall remodeling and accelerates smooth muscle cell migration and proliferation linked to cyclins [25]. Cell proliferation can be promoted through PKN1-cyclin signaling, in which PKN1 is activated by phosphorylation [26]. To explore the mechanism by which ESCs-Exo promote cell proliferation, we examined the phosphorylation of PKN1 and the expression of cyclins. Figure 6 shows that ESCs-Exo can promote the phosphorylation of PKN1 and the expression of cyclins. These findings demonstrated that ESCs-Exo may promote HSFBs proliferation through PKN1-cyclin signaling.

### ESC-Exo application accelerates wound healing

**Figure 7:**
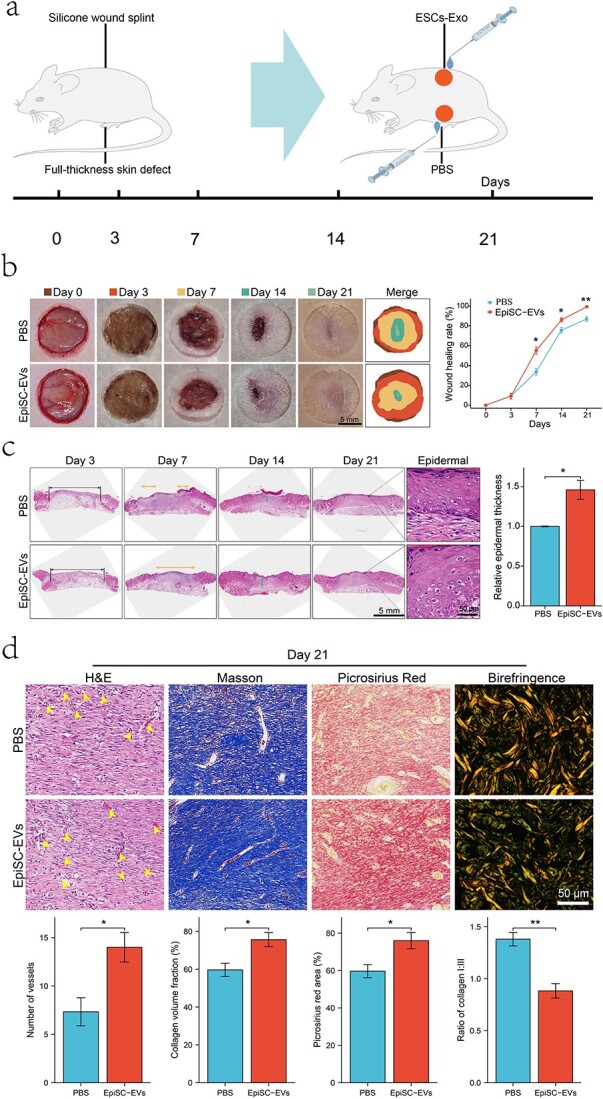
ESCs-Exo promote skin wound healing in mice. (**a**) Animal model. (**b**) Macroscopic wound images and wound healing rates at different time points revealed that ESC-Exos promoted wound healing. (**c**) H&E staining images and epidermal thickness measurements on Day 21. (**d**) Number of blood vessels, collagen content, and type I/III collagen ratio were compared between the two groups by section staining on Day 21, The yellow arrowheads indicates the number of blood vessels. Scale bar: 50μm. The data are presented as the mean ± SD. ^*^p < 0.05, ^*^^*^p < 0.01. *ESCs* pidermal stem cells

To assess the effect of ESCs-Exo on skin wound healing, we monitored changes in the wound status of the mice for 21 days (Figure 7a). Compared with the control group, the Exo group significantly promoted skin wound healing (Figure 7b). The epidermal thickness was evaluated via Hematoxylin and Eosin (H&E) staining at the same time, and the results revealed that, compared with that in the control group, the epidermal thickness of the healed wounds in the experimental group was significantly greater on the 21st day (Figure 7c). Further section staining revealed that the number of blood vessels and collagen and the ratio of type III/I collagen in the Exo group were greater than those in the control group (Figure 7d). In addition, the results of fluorescence staining revealed that Exos promoted the transformation of macrophages from the M1 phenotype to the M2 phenotype (supplementary Fig. 1A, see online supplementary material), reduced the expression of the proinflammatory factors IL-1β and TNF-α, and increased the expression of the anti-inflammatory factor IL-10 (supplementary Fig. 1B). ELISA experiments also confirmed these findings (supplementary Fig. 2, see online supplementary material). These results indicate that ESCs-Exo can promote wound healing and reduce wound inflammation.

### Transcriptomic analysis of ESCs-Exo

**Figure 8:**
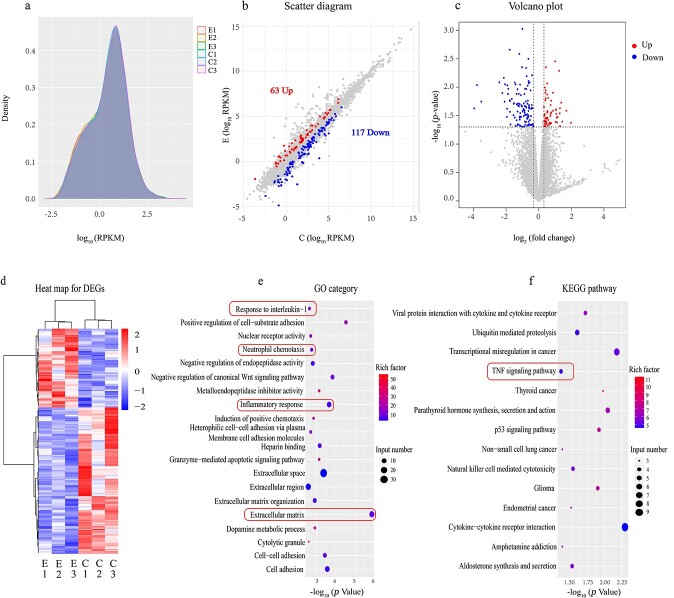
RNA sequencing and functional analyses of the control group and ESCs-Exo. (**a**) Density distribution plot with log10 (RPKM) on the *x*-axis, where a higher value indicates greater gene expression, and gene density on the *y*-axis, representing the number of genes at a given expression level divided by the total number of expressed genes detected. (**b**) Scatter plot with the log2-transformed RPKM values of the ESC-Exo group and control group on the *x*- and *y*-axis, respectively; gray dots represent genes without differential expression, blue dots represent genes with differential downregulation, and red dots represent genes with differential upregulation. (**c**) Volcano plot with the log2-transformed fold-change on the *x*-axis and the negative log10-transformed *P* values on the *y*-axis. (**d**) Clustering heatmap based on RPKM values, where red indicates highly expressed genes and blue indicates genes with low expression. (**e,f**) GO and KEGG enrichment analyses, respectively, where the size of the dot represents the number of DEGs included in the enrichment analysis and the color intensity of the dot indicates the degree of enrichment (rich factor). *ESCs* pidermal stem cells

To further understand the mechanism by which ESCs-Exo promote skin wound healing, we conducted a transcriptomic analysis via high-throughput mRNA sequencing of both the control and ESCs-Exo (40 μg/ml)-treated groups. Initially, we assessed the gene expression levels via reads per kilobase per million reads (RPKM) values as a measure of gene expression [27], with the results presented in the form of density distribution plots (Figure 8a). We set the criteria for differentially expressed genes (DEGs) as those with an absolute log(fold-change) value >0.32 and a *P* value < 0.05, identifying a total of 180 DEGs, of which 117 were downregulated and 63 were upregulated. These DEGs were visualized via scatter plots and volcano plots (Figure 8b and C, respectively). Further clustering analysis of the DEGs was conducted to discern expression patterns under different experimental conditions, revealing significant differential expression. This was visualized via a heatmap (Figure 8d). We subsequently performed Gene Ontology (GO) and Kyoto Encyclopedia of Genes and Genomes (KEGG) enrichment analyses. GO enrichment analysis indicated that ESCs-Exo are associated primarily with factors related to the inflammatory response and components of wound healing, such as the response to IL-1, neutrophil chemotaxis, and the extracellular matrix (Figure 8e). The KEGG enrichment analysis suggested a close relationship between ESCs-Exo and pathways such as the TNF signaling pathway (Figure 8f). ESCs-Exo can modulate the TNF signaling pathway, wherein TNF plays a crucial role in regulating inflammatory and immune responses. During the process of skin wound healing, the TNF signaling pathway is activated to facilitate the inflammatory phase, which is essential for the initial stages of wound healing. This analysis aligns with our previous research findings.

### ESC-Exos regulate the TNF signaling pathway

**Figure 9:**
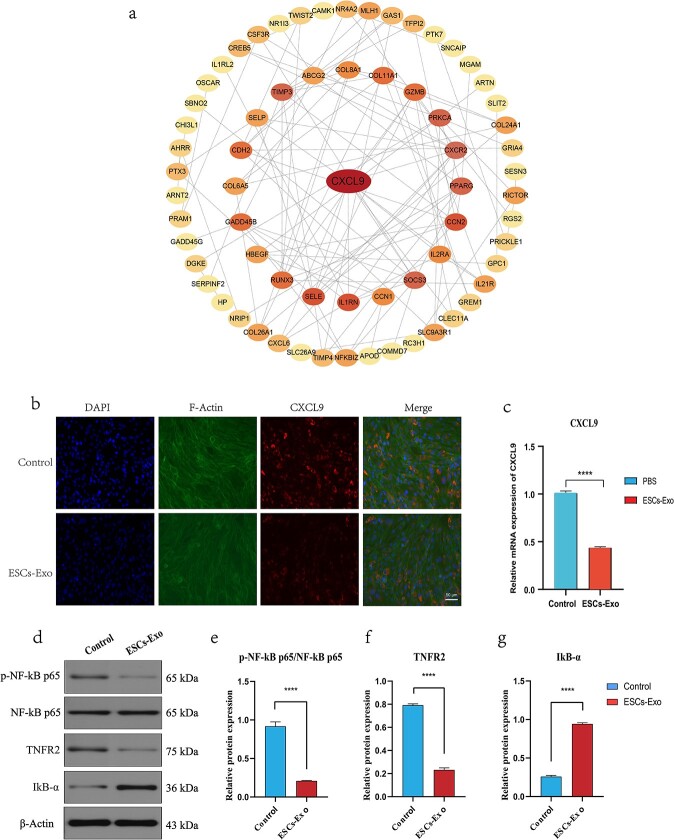
Exos regulate inflammatory factors and inflammatory signaling pathways. (**a**) STRING online website analysis diagram. (**b**) Protein–protein interaction network analysis diagram generated via Cytoscape software. The darker the color, the greater the degree of correlation. (**c**) Immunofluorescence analysis of CXCL9 in the control group and Exo group. Scale bar: 50μm. (**d**) mRNA expression levels of CXCL9 in the control group and Exo group. (**e-h**) Expression levels of phosphorylated (p)-NF-kB p65, NF-kB p65, TNFR2, and IkB-α and quantification. The data are presented as the mean ± SD. ^*^^*^^*^^*^p < 0.001 compared with the control

To study the effects of the 180 differential genes involved in wound healing, these differential genes were imported into the online STRING website (https://cn.string-db.org/) for protein–protein interaction network analysis (Figure 9a). The acquired data were subsequently imported into Cytoscape software (v3.9.0) for further processing and sequencing according to degree, and the most relevant differential gene, C-X-C motif chemokine ligand 9 (CXCL9), was screened, which was used as the Hub gene for subsequent analysis (Figure 9b). CXCL9 is secreted by activated macrophages, endothelial cells, and other immune cells; mediates immune responses primarily by attracting and activating T cells and natural killer cells; and encodes a chemokine protein that plays an important role in inflammatory responses. Both our immunofluorescence and qRT-PCR results revealed that CXCL9 expression was downregulated after Exo treatment compared with that in the control group (Figure 9c and d). We further studied the TNF inflammatory signaling pathway, and the results of western blotting revealed (Figure 9e–h) that the expression of TNFR2 decreased in skin wounds after Exo treatment, which could reduce the level of Nuclear Factor kappa-light-chain-enhancer of activated B cells (NF-kB) phosphorylation and reduce its activity. However, the expression of Inhibitor of kappa B alpha (IkBα) is increased because the anti-inflammatory effect of Exos inhibits IκB Kinase (IKK) activity, thereby reducing the degradation of IkBα. The above results indicate that, after Exo treatment, inflammatory factors and inflammation-related signaling pathways can be regulated, which is also consistent with our previous findings.

## Discussion

Stem cells and related derivatives have been extensively studied in the field of skin wound repair in recent years [28], and the main stem cell types involved include ESCs, MSCs, and ASCs. However, stem cells have drawbacks such as uncertain differentiation, potential tumorigenicity, a low long-term survival rate, and difficulty in preservation and transportation [29], which greatly limits the application of stem cells in the clinical treatment of wounds. Recent evidence suggests that stem cell-derived exosomes have inherently superior tissue repair capabilities [30–32]. Moreover, exosomes have become a focus in the field of wound repair in recent years because of their advantages of easy availability, adequate sources, and low degree of immune rejection [33]. Numerous studies have demonstrated that both MSC-Exos and ASC-Exos improve cutaneous wound healing by affecting all phases of wound healing, including the inflammatory response, cell proliferation, angiogenesis, and collagen synthesis [34, 35]. ESCs are equally popular in regenerative medicine, but studies on the use of ESCs-Exo in wound healing are scarce.

ESCs are self-proliferating and repairing stem cells in the skin that play important roles in the treatment of skin damage caused by burns and various diseases. Stem cells play a repair role through paracrine signals and affect cell proliferation and migration by releasing bioactive factors [36]. Exosomes are important paracrine factors secreted by many cell types and play a leading role in tissue repair and regeneration by transporting functional proteins or miRNAs to neighboring cells as intercellular communication media [37]. The membranes of exosomes are highly similar to those of their parent cells, and stabilizing miRNAs within exosome membranes provides an effective cell-targeted delivery system [38]. Studies have shown that stem cell-derived exosomes can effectively transport mRNAs, miRNAs, proteins, and other bioactive substances, which can not only reduce apoptosis, reduce inflammation, promote angiogenesis, inhibit fibrosis, and improve tissue repair but also have good application prospects in the regulation of tissue regeneration [39, 40]. Compared with ESCs, ESCs-Exo are easier to obtain, are nonimmunogenic, and can penetrate the cell membrane to play a role in the cell [9].

The main functional cells involved in wound healing and scar remodeling are fibroblasts, which proliferate, migrate to the wound, and secrete collagen fibers to seal and protect the wound. After injury, fibrin clots form rapidly to prevent thrombus. Platelets in the blood trigger a clotting cascade and secrete a variety of growth factors that promote wound healing [41]. During the subsequent inflammatory phase, neutrophils migrate to the wound, kill bacteria, engulf foreign debris via phagocytosis, and release proteolytic enzymes. Moreover, monocytes infiltrate the injury site and differentiate into macrophages, releasing a mixture of proteases and bioactive molecules, including transforming growth factor β1, which stimulates the migration of fibroblasts and epithelial cells [42,43]. The proliferative phase usually begins ~3 days after injury and includes angiogenesis (via endothelial cells), granulation tissue formation (via fibroblasts), and re-epithelialization (via keratinocytes). At this stage, fibroblasts produce large amounts of extracellular matrix, mainly collagen, to form granulation tissue and cover the damaged tissue. At the same time, keratinocytes migrate, proliferate, differentiate, and reform a functional epidermis (re-epithelialization), protecting the tissue from further damage. As the wound matures, the granulation tissue is replaced by dermal fibroblasts. Therefore, studying the proliferation and migration of fibroblasts for wound healing is highly important [44, 45].

In this study, we efficiently generated, isolated, and characterized ESC-Exos and explored their optimal concentration and functions via *in vitro* assays of differentiation, migration, and proliferation. Our findings show that ESCs-Exo can promote the proliferation and migration of HSFBs. ESCs-Exo significantly enhanced the proliferation of HSFBs in a dose-dependent manner. Moreover, ESCs-Exo greatly enhanced the movement of HSFBs in a dose-dependent manner. They all reached a plateau at 40 μg/ml, suggesting that this could be the optimal concentration for the beneficial effects of ESCs-Exo. Moreover, we examined the phosphorylation of PKN1 and the expression of cyclins. The results show that ESCs-Exo can promote the phosphorylation of PKN1 and the expression of cyclins. These findings indicate that ESC-Exos may promote HSFB proliferation through PKN1-cyclin signaling. *In vivo* animal experiments revealed that ESCs-Exo significantly promoted the healing of skin wounds, accelerated the formation of blood vessels and collagen, promoted the transformation of macrophages from the M1 type to the M2 type, and reduced the inflammatory response of wounds. We further performed transcriptomic analysis on the wound tissue, and functional enrichment analysis revealed that the DEGs were closely related mainly to inflammatory factors and the TNF inflammatory signaling pathway. We subsequently identified the key gene CXCL9 and verified the phenotypic functions of the core members of the CXCL9 and TNF signaling pathways through phenotypic function experiments. The results revealed that ESCs-Exo significantly reduced the inflammatory response compared with that in the control group, and interestingly, these findings are consistent with our results in animal experiments.

The collagen composition of skin is known to be mainly collagen I and collagen III, and the proportion of collagen I/III changes with age. The ratio of collagen I/III content in the skin of 15-week-old fetuses was 0.8 : 1, whereas it was 3.6 : 1 at 3 months after birth, which is close to the ratio of adults (3.5–6 : 1). The proportion of collagen III in keloids is 1 : 19, and the content of collagen III is much lower than that in hypertrophic scars and lower than that in normal skin [46–48]. In our experiments, we found that treating HSFBs with ESCs-Exo effectively increased the ratio of collagen III/I. These findings suggest that ESCs-Exo may have antiscarring potential.

## Conclusion

In this study, we found that ESCs-Exo can promote the proliferation and migration of HSFBs, which may be the mechanism by which exosomes accelerate wound healing. In addition, ESCs-Exo may have value in the prevention or treatment of abnormal scars. Considering the limited scalability and immunological problems associated with ESCs, our strategy may have the potential to produce exosomes to replace ESCs for trauma therapy.

## Abbreviations

DAPI: Diamidinyl phenyl indole; DEGs: Differentially expressed genes; ELISA: Enzyme-linked immunosorbent assay; ESCs: Epidermal stem cells; ESC-Exos: Epidermal stem cell-derived exosomes; FBS: Fetal bovine serum; HSFBs: Human skin fibroblasts; IL-1: Interleukin 1; PBS: Phosphate-buffered saline; RPKM: Reads per kilobase per million reads; SD: Standard deviation; TNF: Tumor necrosis factor.

## Supplementary Material

Supplementary_Figure_1_tkae047

Supplementary_Figure_2_tkae047

## Data Availability

The data that support the findings of this study are available from the corresponding author upon reasonable request.
