# Supplementary figures and images for: Epidermal stem cell-derived exosomes improve wound healing by promoting the proliferation and migration of human skin fibroblasts
Source: Burns Trauma. 2024 Dec 16;12:tkae047. doi: 10.1093/burnst/tkae047 (PMC11647520; doi:10.1093/burnst/tkae047)

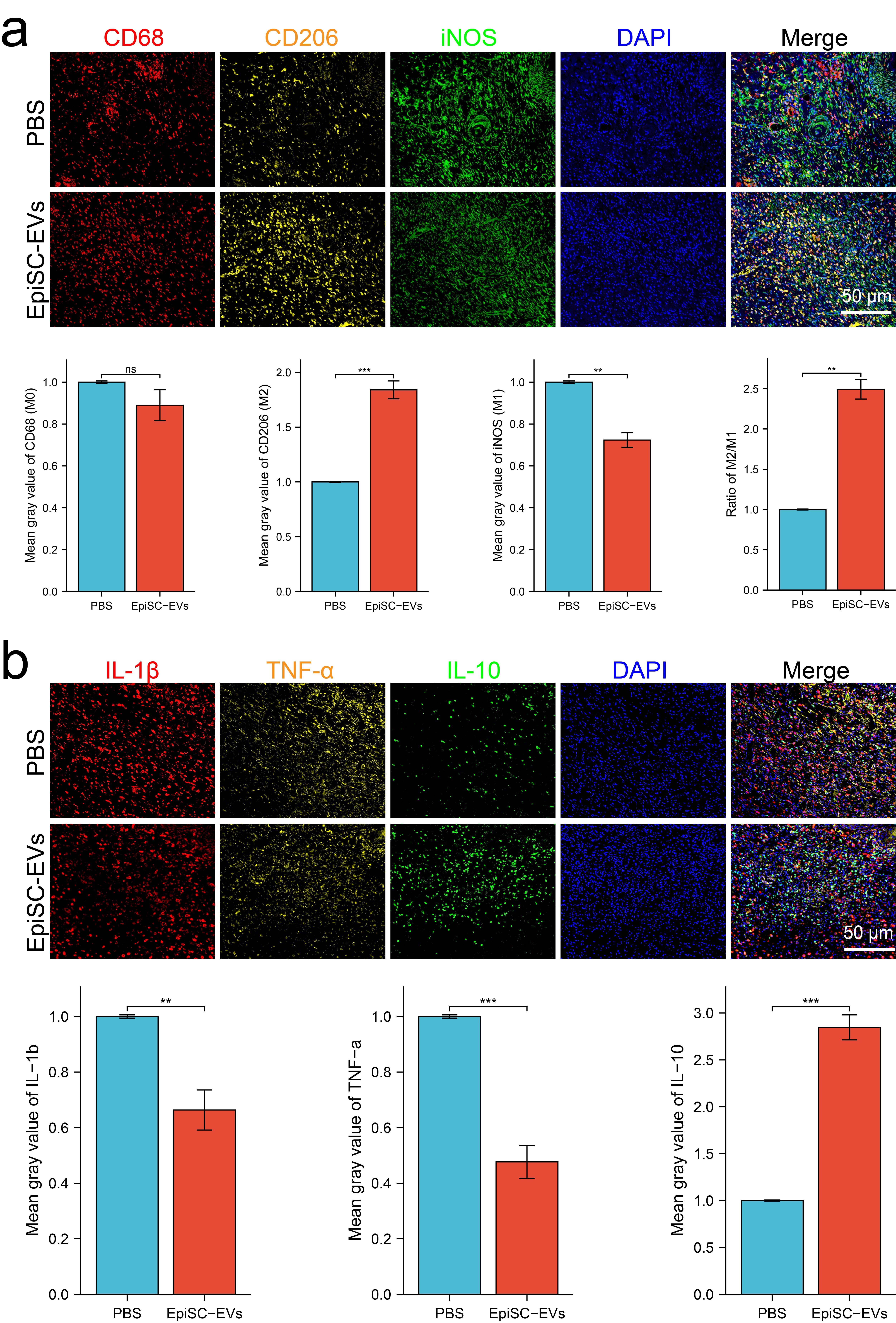

Supplement: Supplementary_Figure_1_tkae047 [file supplementary_figure_1_tkae047.jpeg]

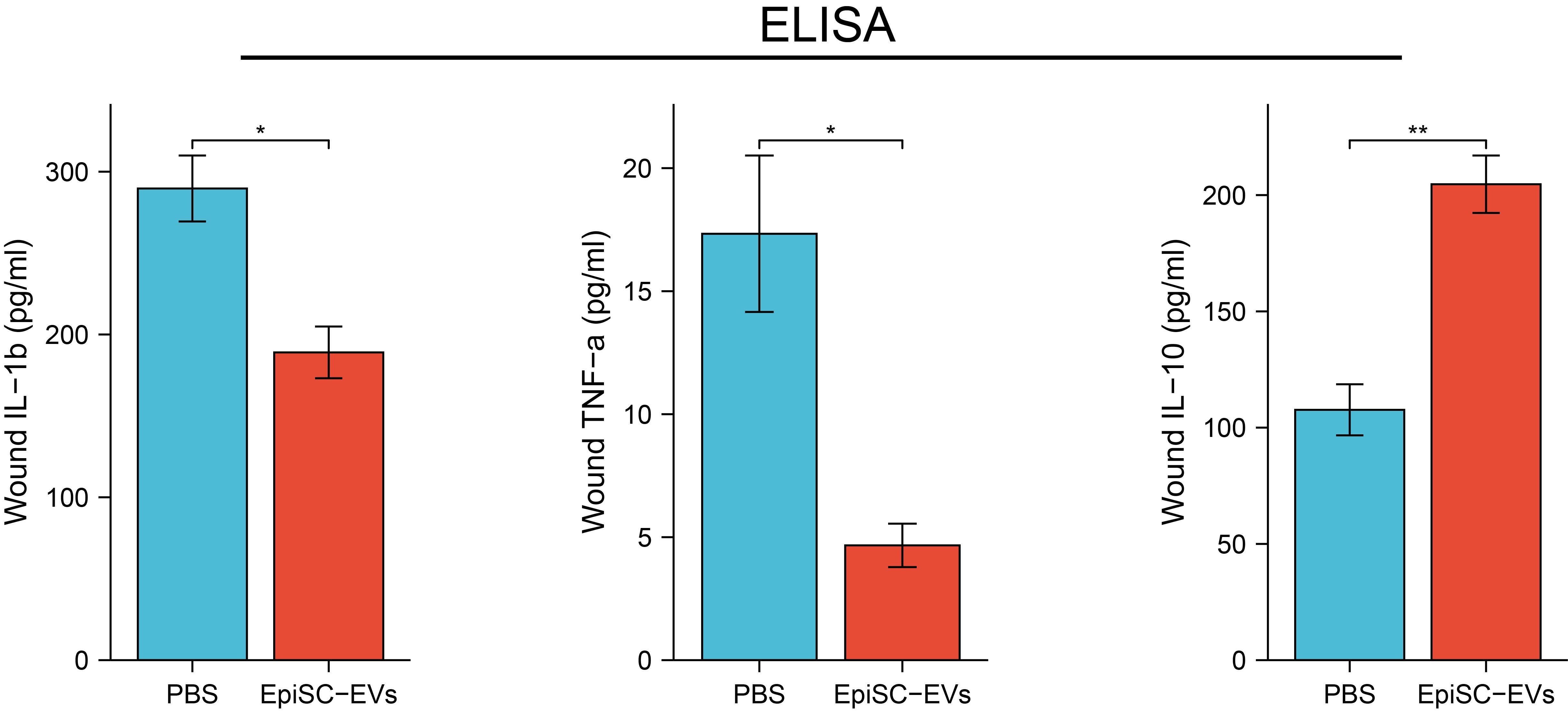

Supplement: Supplementary_Figure_2_tkae047 [file supplementary_figure_2_tkae047.jpeg]
